# Genetically Proxied GIP‐GIPR Pathway Perturbation and Fracture Risk

**DOI:** 10.1111/dom.71378

**Published:** 2026-09-29

**Authors:** Wenjie Cheng, Weijie Liu, Jie Wei, Chao Zeng, Houchen Lyu, Sizheng Steven Zhao

**Affiliations:** ^1^ Centre for Musculoskeletal Research, Division of Musculoskeletal and Dermatological Science, School of Biological Sciences, Faculty of Biological Medicine and Health The University of Manchester, Manchester Academic Health Science Centre Manchester UK; ^2^ Dermatology Center Xinhua Hospital, Shanghai Jiaotong University School of Medicine Shanghai Shanghai China; ^3^ Department of Epidemiology and Health Statistics, Xiangya School of Public Health Central South University Changsha Hunan China; ^4^ Health Management Center Xiangya Hospital, Central South University Changsha Hunan China; ^5^ Department of Orthopaedics Xiangya Hospital, Central South University Changsha Hunan China; ^6^ Department of Orthopaedics The Chinese PLA General Hospital Beijing Beijing China; ^7^ National Clinical Research Center for Orthopedics and Sports Medicine The Chinese PLA General Hospital Beijing Beijing China; ^8^ NIHR Manchester Biomedical Research Centre Manchester University NHS Foundation Trust Manchester UK

**Keywords:** cohort study, incretin therapy, risk prediction, Type 2 diabetes

## Abstract

**Aims:**

To determine whether genetic proxies for incretin pathway perturbation are associated with fracture risk and bone mineral density, providing human genetic evidence relevant to the skeletal safety of incretin‐based therapies.

**Materials and Methods:**

We conducted drug‐target Mendelian randomisation using cis genetic variants near GIP, GIPR, GCG, GLP1R in 401 983 unrelated UK Biobank participants. Instruments were scaled to 1 kg/m^2^ lower BMI or 1 mmol/mol lower HbA1c. Outcomes included fractures at major osteoporotic sites, combined spine‐and‐hip fractures, site‐specific fractures and estimated bone mineral density (eBMD). We compared these estimates with genome‐wide liability to lower BMI or HbA1c to contextualise the fracture associations observed for the target‐proximal instruments. We used Cox models to estimate time‐to‐fracture associations per SD of target genetic risk score aligned to lower BMI or HbA1c.

**Results:**

GIP‐GIPR perturbation was associated with lower odds of fractures at major osteoporotic sites when scaled to lower BMI (OR 0.80, 95% CI 0.66–0.98) or lower HbA1c. Associations were similar for combined spine‐and‐hip fractures under both scaling approaches (OR 0.68, 0.49–0.94). Genome‐wide liability to lower BMI or HbA1c did not show the same fracture associations as GIP‐GIPR perturbation. GCG‐GLP1R estimates were imprecise. Genetically proxied incretin pathway perturbation was not associated with eBMD. In Cox analyses, GIP‐GIPR genetic risk score was associated with lower risk of fractures at major osteoporotic sites (HR 0.976, 0.959–0.992) and combined spine‐and‐hip fractures (HR 0.971, 0.946–0.996).

**Conclusions:**

Human genetic evidence suggests an association between genetically proxied GIP‐GIPR pathway perturbation and lower fracture risk, supporting fracture‐focused evaluation of GIPR‐targeting therapies in clinical and post‐marketing studies.

## 1 Introduction

Incretin‐based therapies are rapidly reshaping the management of Type 2 diabetes and obesity, exposing millions of individuals to sustained GLP‐1 receptor and, increasingly, GIP/GLP‐1 receptor activation [1, 2]. As these agents expand into broader metabolic disease populations, their safety has become increasingly important [3, 4]. One clinically relevant but unresolved question is whether long‐term perturbation of incretin signalling influences skeletal health and fracture risk.

Existing evidence has not resolved whether incretin therapies increase, decrease, or have no meaningful effect on fracture risk [5, 6]. Randomised trials have generally not been designed or powered to assess fracture outcomes [7, 8], and observational studies can be challenging to interpret because users differ in characteristics that are also important for fracture risk [9, 10, 11]. These limitations are particularly relevant for newer agents targeting the glucose‐dependent insulinotropic polypeptide (GIP) receptor and for dual GIPR/GLP‐1 receptor (GLP1R) agonists, where clinical use is expanding but long‐term skeletal safety data are sparse. Mechanistically, the direction of effect is uncertain: improvements in glycaemic control and reductions in inflammation could plausibly benefit skeletal health [6, 12], whereas loss of lean mass may increase susceptibility to falls and fractures [13, 14].

Human genetics offer a complementary way to address this question [15]. Genetic variations in genes encoding incretin pathway drug targets can proxy their lifelong perturbation and enable assessment of fracture outcomes with less susceptibility to confounding by indication than conventional observational studies [16]. We therefore examined whether genetically proxied perturbation of incretin signalling—focusing on the GIP‐GIPR and GCG‐GLP1R pathways—was associated with fracture risk and bone mineral density.

## 2 Materials and Methods

### 2.1 Study Overview

We conducted a drug‐target Mendelian randomisation (MR) study using genetic variants in or near the genes encoding incretin ligands and receptors (combined into a genetic risk score, GRS) to proxy perturbation of incretin signalling. The overall study design is shown in Figure 1. Focusing on downstream biomarkers (BMI and HbA1c), we tested associations with fracture outcomes and estimated heel bone mineral density (eBMD) and performed complementary GRS‐based time‐to‐event analyses using individual‐level UK Biobank data. Where possible, we attempted validation using two‐sample MR with summary statistics from FinnGen [17] (freeze 12) and the Million Veteran Program [18] (MVP).

**FIGURE 1:**
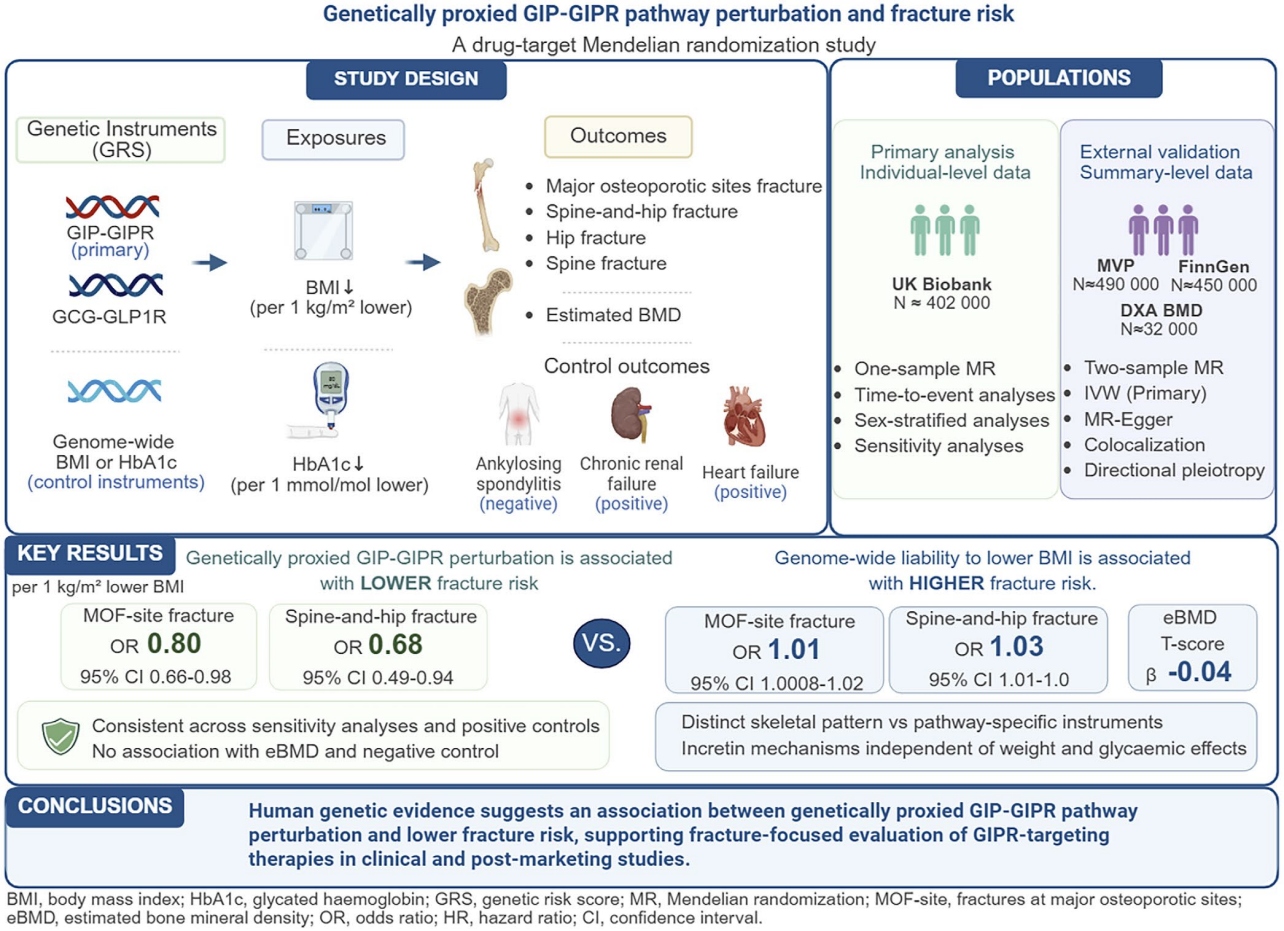
Study design overview. This study used a drug‐target MR framework to investigate the effects of genetically proxied perturbation of incretin signalling on fracture outcomes. GRS were constructed using cis‐variants in or near genes encoding incretin pathway components, including GIP‐GIPR and GCG‐GLP1R, and aligned to lower BMI or lower HbA1c as downstream phenotypic measures of target perturbation. These instruments were used to estimate associations with MOF‐site fractures, spine‐and‐hip fractures, site‐specific fractures and eBMD. Analyses were conducted primarily in UK Biobank using individual‐level data, with external validation using summary‐level data from the MVP, FinnGen and DXA‐BMD datasets. Analyses included one‐sample MR, two‐sample MR and time‐to‐event analyses, alongside sensitivity analyses using alternative and missense‐variant instruments. BMI, body mass index; DXA, dual‐energy X‐ray absorptiometry; eBMD, estimated bone mineral density; GRS, genetic risk score; HbA1c, glycated haemoglobin; MOF‐site, site‐based major osteoporotic fracture phenotype; MR, Mendelian randomisation; MVP, Million Veteran Program; UKB, UK Biobank.

### 2.2 UK Biobank Participants

UK Biobank recruited approximately 500 000 individuals aged 40–69 years between 2006 and 2010, with linked health outcome data [19]. We restricted analyses to unrelated participants (kinship coefficient < 0.0884) of White British ancestry with concordant genetic and self‐reported sex. The final analytic sample included 401 983 individuals. Each analysis included the largest subset of participants with complete data for the relevant exposure and outcome.

### 2.3 Genotyping, Imputation and Genetic Quality Control

Genotyping was performed centrally by UK Biobank using the UK BiLEVE Axiom array for the initial subset and the UK Biobank Axiom array for the remainder, with standard sample‐ and variant‐level quality control conducted by UK Biobank [19]. UK Biobank phased and imputed genotypes using reference panels including the Haplotype Reference Consortium (HRC) and a merged UK10K/1000 Genomes reference where required. In this study, we restricted variants to those with imputation INFO score > 0.9 and minor allele frequency > 1%.

### 2.4 Genetic Instruments for Incretin Signalling

Cis genetic instruments were constructed using variants within 250 kb of genes encoding incretin ligands and receptors, including glucose‐dependent insulinotropic polypeptide (*GIP*), its receptor (*GIPR*), proglucagon (*GCG*) and the glucagon‐like peptide‐1 receptor (*GLP1R*). GLP1 is a cleavage product of proglucagon encoded by the *GCG* gene, thus variants near *GCG* were used to proxy upstream GLP1 ligand biology. Variants were selected from external European‐ancestry GWAS of BMI [20] or HbA1c [21], prioritising datasets without UK Biobank overlap to minimise winner's curse. Independent variants (*r* ^2^ < 0.1) [22] reaching genome‐wide significance were used where available; otherwise variants reaching regional significance (*p* ≤ 5 × 10^−6^) [23, 24, 25] were used if instrument strength was adequate based on F‐statistics. Variants with opposing effects on BMI and HbA1c (*p* < 0.05) were excluded to avoid combining variants with discordant downstream metabolic associations and to preserve a consistent phenotypic orientation of the genetic instrument.

Primary instruments were constructed for two pathways: GIP‐GIPR and GCG‐GLP1R. For GIP‐GIPR, two variants (rs35895680 and rs2302593) were selected for the primary instrument. Given the small number of variants, each was also analysed separately to assess the contribution of the individual variants to the observed associations. Additional variants, including missense variants, were evaluated. For GCG‐GLP1R, no variants met the prespecified criteria in external datasets; selection was therefore extended to GWAS including UK Biobank data using a relaxed threshold (*p* ≤ 5 × 10^−5^) [26, 27]. Detailed characteristics of the selected variants, including the source datasets, discovery *p*‐values and UK Biobank association statistics, are provided in Table S1.

GRS were constructed by summing effect alleles. Alleles were aligned according to their downstream phenotypic association, such that the effect allele corresponded to lower BMI or lower HbA1c, and genotypes were coded as 0, 1 or 2 copies. Unweighted GRS were used for the primary instruments. Conditional weighting was applied only for the GIP‐GIPR sensitivity score containing variants in modest LD (*r* ^2^ = 0.185), using adjusted *β* coefficients from linear regression of the exposure on each variant, adjusting for the remaining variants in the score. Instrument strength was assessed using the *F* statistic [28], with *F* > 10 indicating adequate strength. Details of GRS construction are provided in Table S2.

Body mass index (BMI, kg/m^2^) was calculated from measured height and weight at baseline. Glycated haemoglobin (HbA1c, mmol/mol) was measured using high‐performance liquid chromatography (VARIANT II Turbo, Bio‐Rad). Implausible values were excluded (BMI < 10 or > 60 kg/m^2^; HbA1c outside 15–184 mmol/mol). Genetic effects were scaled to BMI or HbA1c to provide clinically interpretable measures of the genetically proxied pathway perturbation.

### 2.5 Outcomes

Fracture outcomes were derived from linked hospital admission data and mortality records using International Classification of Diseases, 10th revision (ICD‐10) codes [29, 30, 31] (Table S3). The primary outcome was a site‐based major osteoporotic fracture phenotype (MOF‐site), defined as fractures at the thoracic or lumbar spine, hip/femur, pelvis, humerus or forearm, together with selected vertebral collapse, fatigue fracture codes and osteoporosis‐with‐pathological‐fracture codes at corresponding sites. This phenotype was not intended to distinguish fractures by trauma mechanism and therefore may include both low‐ and high‐trauma fractures. Secondary outcomes included combined spine‐and‐hip fracture, isolated spine fracture and isolated hip fracture. The combined spine‐and‐hip fractures were defined to increase statistical power, and these secondary outcomes are partially overlapping. eBMD was measured by calcaneal quantitative ultrasound in approximately half of participants at baseline and reported as sex‐matched T‐scores in standard deviation (SD) units.

### 2.6 Control Outcomes

As a prespecified assessment of instrument performance, we examined positive control outcomes (heart failure and chronic renal failure) based on established effects of incretin therapies [3, 32, 33], and a negative control outcome (ankylosing spondylitis) with no known mechanistic link to incretin signalling. The prespecified interpretive hierarchy required an exposure *F*‐statistic > 10, directionally concordant associations with both positive controls reaching nominal significance (*p* < 0.05), and no statistical evidence of association with the negative control (*p* > 0.05) for an instrument to be prioritised for primary interpretation. Instruments not meeting these criteria were reported in full but interpreted as exploratory. These control analyses were intended to assess consistency with the expected downstream phenotype rather than to establish pharmacological equivalence or exclude pleiotropy.

### 2.7 Statistical Analysis

#### 2.7.1 One‐Sample Mendelian Randomisation

All analyses were conducted in R (version 4.5.1). One‐sample MR used the ratio approach. The unweighted GRS was treated as a single score‐level genetic instrument. For each GRS, associations with binary outcomes (fractures and controls) were estimated using logistic regression and associations with eBMD were estimated using linear regression. The corresponding GRS‐exposure association was estimated using linear regression. Models were adjusted for age, sex (where applicable) and PC1‐PC10. The ratio estimate was computed as β(GRS → outcome)/β(GRS → exposure). For binary outcomes, the logistic‐regression coefficient on the log‐odds scale was divided by the GRS‐exposure coefficient; the resulting ratio estimate was exponentiated and reported as an odds ratio per 1 kg/m^2^ lower BMI or per 1 mmol/mol lower HbA1c. Standard errors were obtained using the delta method.

Sex‐stratified analyses were performed as descriptive analyses. GRS‐by‐sex interaction terms were included in regression models to formally assess sex‐dependent effects, with interaction *p*‐values reported in Table S4.

#### 2.7.2 Time‐To‐Event Analyses

Cox proportional hazards models were used to estimate associations with incident fracture outcomes. Participants entered the risk set at their UK Biobank baseline assessment date. Individuals with a relevant fracture code before baseline were excluded from the corresponding incident outcome analyses. Follow‐up commenced at baseline and continued until the first fracture event, death, the last valid follow‐up observation, or the administrative end of linked Hospital Episode Statistics (HES) data (April 2022), whichever occurred first. Models were adjusted for age, sex and PC1‐PC10, with analyses repeated in sex‐stratified models. Each GRS was standardised to z‐scores.

Absolute risk differences at 10 years corresponding to a 5 kg/m^2^ reduction in BMI or a 5 mmol/mol reduction in HbA1c were estimated by applying hazard ratios to observed baseline cumulative incidence. To account for competing risks, we performed complementary analyses using the Aalen‐Johansen (AJ) model.

#### 2.7.3 External Validation and Sensitivity Analyses

Two‐sample MR analyses were conducted using fracture outcome summary statistics from FinnGen [17] and MVP [18], with DXA‐measured BMD [34] expressed as SD units additionally included in analyses (Table S5). Fixed‐effect inverse‐variance weighted models were used as the primary approach, with MR‐Egger as a sensitivity analysis. Directional pleiotropy was assessed using the MR‐Egger intercept. Multiple testing was addressed using false discovery rate (FDR) correction across outcomes for each instrument.

Genetic instruments were derived from BMI [26] and HbA1c [27] GWAS. Cis instruments were selected within ±250 kb of target loci (*p* < 5 × 10^−8^, *r* ^2^ < 0.1). Instruments prioritised ligand‐receptor perturbation where possible (e.g., GIP‐GIPR). Genome‐wide comparator variants outside these regions were selected at *p* < 5 × 10^−8^ and pruned using *r* ^2^ < 0.001.

Colocalization analyses were performed for associations with IVW *p* < 0.05 within ±50 kb of the cis‐region using default priors (p1 = 1 × 10^−4^, p2 = 1 × 10^−4^, p12 = 1 × 10^−5^), with strong evidence defined as P4/(P3 + P4) > 0.8. Analyses were conducted in R using the TwoSampleMR [35], MendelianRandomisation [36] and coloc [37] packages.

Sensitivity analyses also included missense variants used in published studies, some of which were not included in the primary instruments, for example, because of a minor allele frequency < 1% [24, 25, 32] (Table S6). Genome‐wide polygenic risk scores (PRS) for BMI and HbA1c were additionally included as contextual comparators, derived from external GWAS and calculated in UK Biobank participants.

## 3 Results

### 3.1 Associations of Incretin Pathway Instruments With Control Outcomes

Analyses included 401 983 unrelated White British participants from the UK Biobank (Table S7).

The GIP‐GIPR instrument showed the most consistent evidence across positive control outcomes. The HbA1c‐scaled GCG‐GLP1R instrument showed partial concordance with positive controls, whereas the BMI‐scaled GCG‐GLP1R instrument showed inconsistent signals across control outcomes. The GCG‐GLP1R analyses were therefore considered exploratory.

Genetically proxied GIP‐GIPR perturbation showed protective associations with both positive controls when scaled to BMI (heart failure OR 0.67, 95% CI 0.52 to 0.86; chronic renal failure OR 0.73, 0.58 to 0.92) and when scaled to HbA1c (heart failure OR 0.68, 0.53 to 0.87; chronic renal failure OR 0.74, 0.59 to 0.93), with no evidence of association with the negative control (OR 1.33, 0.56 to 3.17 for BMI scaling; OR 1.42, 0.61 to 3.30 for HbA1c scaling) (Table S8).

Genetically proxied GCG‐GLP1R perturbation showed partial concordance across positive controls: HbA1c‐scaled estimates indicated lower odds of chronic renal failure (OR 0.76, 0.64 to 0.92), whereas estimates for heart failure lacked precision (OR 0.90, 0.74 to 1.09), and BMI‐scaled estimates had wide confidence intervals across all control outcomes.

### 3.2 GIP‐GIPR Pathway Perturbation Scaled to Lower BMI Is Associated With Lower Fracture Risk at Osteoporotic Sites

Genetic perturbation of the GIP‐GIPR pathway scaled to 1 kg/m^2^ lower BMI was associated with lower odds of ICD‐coded fractures at major osteoporotic sites (MOF‐site events) (OR 0.80, 0.66 to 0.98) (Figure 2A). Protective associations were also observed for secondary fracture outcomes, including combined spine‐and‐hip fractures (OR 0.68, 0.49 to 0.94) and spine fractures (OR 0.60, 0.36 to 0.99), whereas estimates for hip fractures were directionally similar but imprecise (OR 0.72, 0.48 to 1.08) (Table S9). These secondary outcomes were interpreted as supportive findings rather than independent confirmatory endpoints.

**FIGURE 2:**
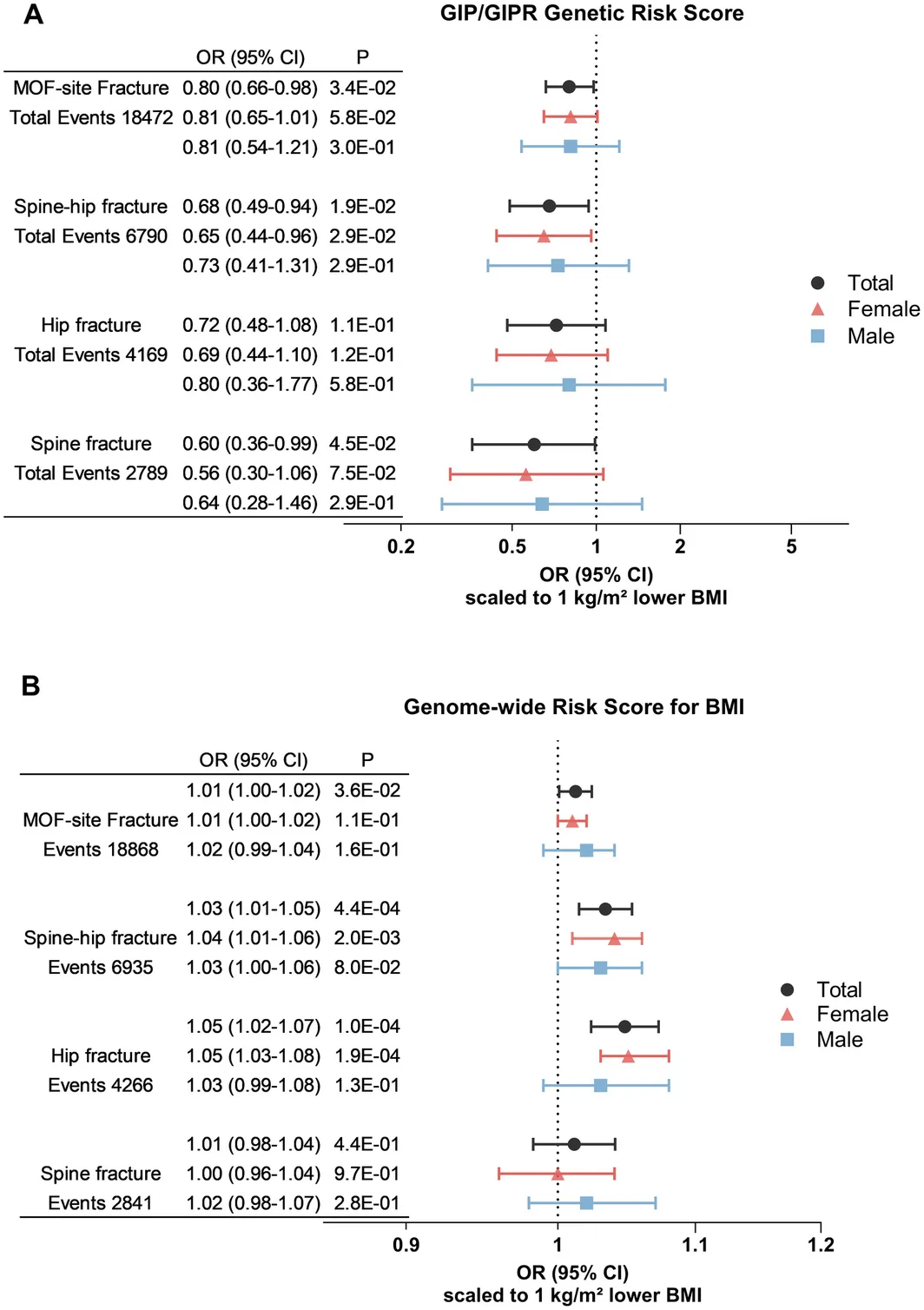
Fracture risk associated with genetically proxied GIP‐GIPR perturbation and genetic liability to lower BMI. One‐sample Mendelian randomisation analyses in UK Biobank showing odds ratios for fractures at major osteoporotic sites, combined spine‐and‐hip fractures, spine fractures and hip fractures per 1 kg/m^2^ lower BMI proxied by the primary unweighted GIP‐GIPR GRS (A) or a genome‐wide BMI polygenic score (B). Sex‐stratified analyses are shown for the overall population (circles), females (triangles) and males (squares). Analyses were restricted to unrelated White British participants. Error bars indicate 95% confidence intervals. BMI, body mass index; CI, confidence interval; GRS, genetic risk score.

Sex‐stratified analyses yielded more precise estimates in females than in males. Among females, the BMI‐scaled GIP‐GIPR perturbation showed directionally consistent estimates for combined spine‐and‐hip fractures (OR 0.65, 0.44 to 0.96) and MOF‐site events (OR 0.81, 0.65 to 1.01) (Table S10). In single‐variant analyses, the GIP variant was nominally associated with lower odds of the primary MOF‐site outcome and with the positive control outcomes, whereas the GIPR variant produced directionally concordant but less precise estimates. Alternative GIP‐GIPR instrument specifications also showed broadly concordant associations with fracture outcomes (Tables S11 and S12).

No association was observed with estimated bone mineral density (eBMD) measured by calcaneal ultrasound (*β* 0.01 T‐score units per 1 kg/m^2^ lower BMI, −0.08 to 0.05) (Table S13).

### 3.3 GIP‐GIPR Pathway Perturbation Scaled to Lower HbA1c Is Associated With Lower Fracture Risk at Osteoporotic Sites

Genetic perturbation of the GIP‐GIPR pathway scaled to 1 mmol/mol lower HbA1c was associated with lower odds of MOF‐site events (OR 0.80, 0.65 to 0.98) (Figure 3A). Protective associations were also observed for combined spine‐and‐hip fractures (OR 0.68, 0.49 to 0.94) and spine fracture (OR 0.57, 0.35 to 0.95). Estimates for hip fractures were directionally similar but imprecise (OR 0.75, 0.50 to 1.13) (Table S14).

**FIGURE 3:**
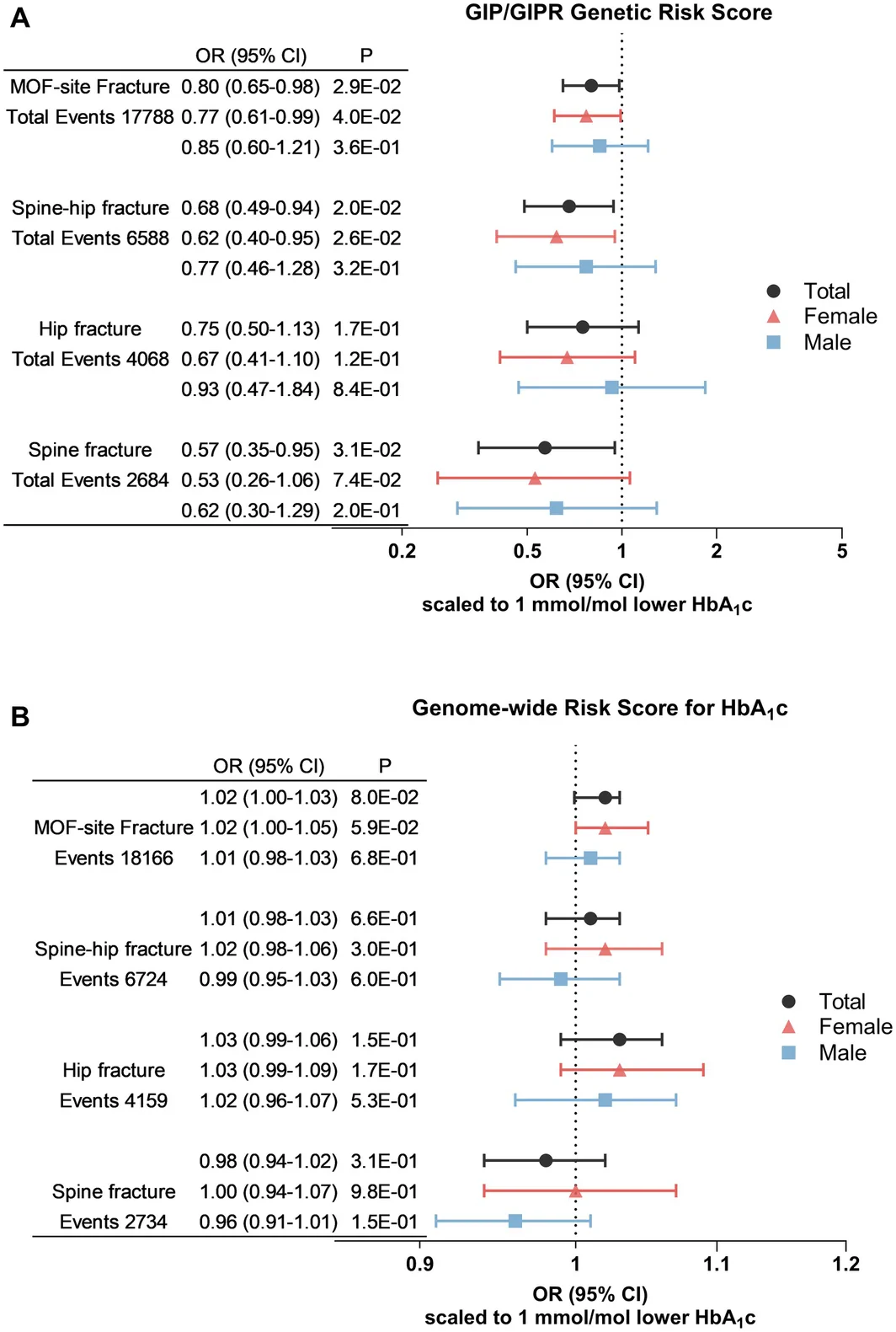
Fracture risk associated with genetically proxied GIP‐GIPR perturbation and genetic liability to lower HbA1c. One‐sample Mendelian randomisation analyses in UK Biobank showing odds ratios for fractures at major osteoporotic sites, combined spine‐and‐hip fractures, spine fractures and hip fractures per 1 mmol/mol lower HbA1c proxied by the primary unweighted GIP‐GIPR GRS (A) or a genome‐wide HbA1c polygenic score (B). Sex‐stratified analyses are shown for the overall population (circles), females (triangles) and males (squares). Analyses were restricted to unrelated White British participants. Error bars indicate 95% confidence intervals. CI, confidence interval; HbA1c, glycated haemoglobin; GRS, genetic risk score.

Effect estimates were more precise in females. HbA1c‐scaled GIP‐GIPR perturbation was associated with lower odds of MOF‐site events (OR 0.77, 0.61 to 0.99) and combined spine‐and‐hip fractures in females (OR 0.62, 0.40 to 0.95) (Table S15). Sensitivity analyses using alternative GIP‐GIPR instrument specifications yielded directionally concordant estimates across fracture outcomes, with the association for combined spine‐and‐hip fractures reaching nominal statistical significance (OR 0.73, 0.55 to 0.97).

No association was observed with eBMD (*β* −0.02 T‐score units per 1 mmol/mol lower HbA1c, −0.09 to 0.04) (Table S16).

### 3.4 Exploratory Analyses of GCG‐GLP1R Pathway Perturbation Show Less Consistent Associations Across Fracture Outcomes

Genetically proxied perturbation of the GCG‐GLP1R pathway showed less consistent associations across fracture outcomes (Tables S9 and S10, S14 and S15). HbA1c‐scaled analyses suggested lower odds for some endpoints, particularly spine fracture (OR 0.61, 0.40 to 0.91), but estimates for MOF‐site events (OR 0.85, 0.73 to 1.01), combined spine‐and‐hip fractures (OR 0.82, 0.63 to 1.06) and hip fractures (OR 0.99, 0.71 to 1.37) were less precise and not consistently supported across control outcomes and sensitivity analyses (Figure 4).

**FIGURE 4:**
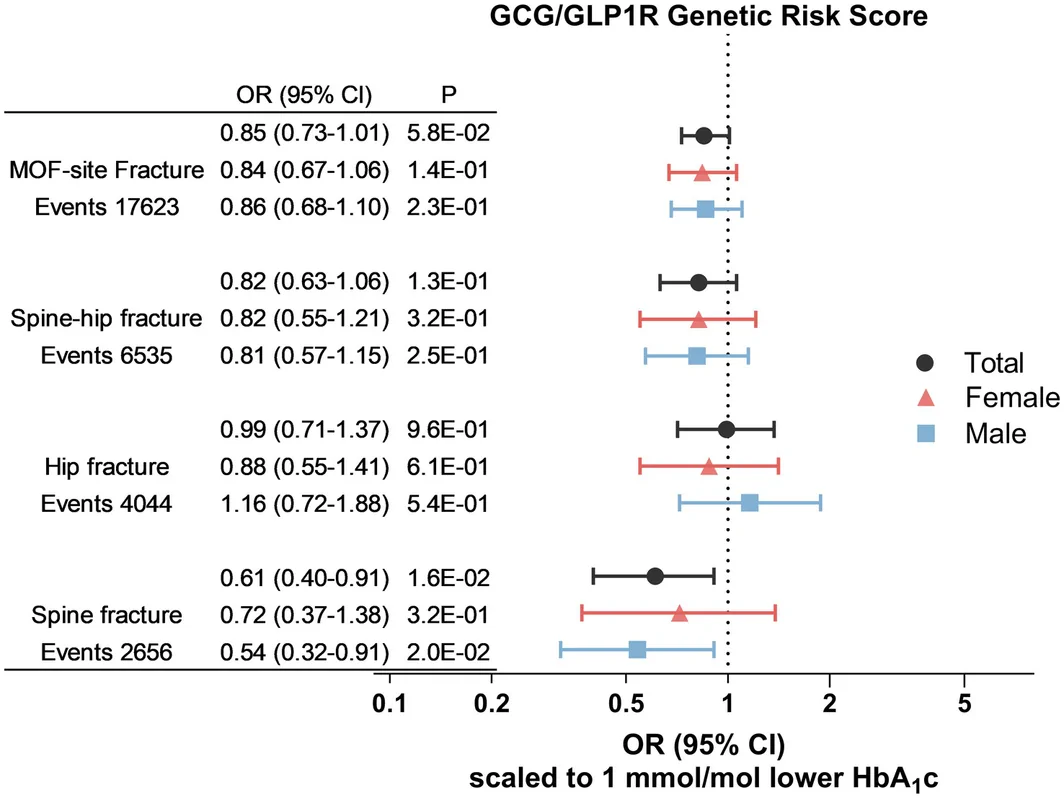
Fracture risk associated with genetically proxied GCG‐GLP1R perturbation to lower HbA1c. One‐sample Mendelian randomisation analyses in UK Biobank showing odds ratios for fractures at major osteoporotic sites, combined spine‐and‐hip fractures, spine fractures and hip fractures per 1 mmol/mol lower HbA1c proxied by the unweighted GCG‐GLP1R GRS. Sex‐stratified analyses are shown for the overall population (circles), females (triangles) and males (squares). Analyses were restricted to unrelated White British participants. Error bars indicate 95% confidence intervals. CI, confidence interval; GRS, genetic risk score; HbA1c, glycated haemoglobin.

Sex‐stratified estimates were generally imprecise. No association was observed with eBMD (*β* −0.02 T‐score units per 1 mmol/mol lower HbA1c, −0.07 to 0.03).

For BMI‐scaled GCG‐GLP1R perturbation, estimates had wide confidence intervals across most fracture and control outcomes (e.g., MOF‐site fractures OR 0.91, 0.68 to 1.23; heart failure OR 0.70, 0.49 to 1.02), except for a protective effect on spine fractures (OR 0.45, 0.21 to 0.99). These results should be interpreted cautiously. No association was observed with eBMD (*β* −0.05 T‐score units per 1 kg/m^2^ lower BMI, −0.15 to 0.04).

### 3.5 Genome‐Wide BMI and HbA1c Liability Show Different Skeletal Association Patterns From Pathway‐Scaled Instruments

By contrast, genetic liability to lower BMI showed strong associations with higher fracture risk, including MOF‐site events (OR 1.01, 1.0008 to 1.02), combined spine‐and‐hip fractures (OR 1.03, 1.01 to 1.05), and hip fractures (OR 1.05, 1.02 to 1.07), and lower eBMD (*β* −0.04 T‐score units per 1 kg/m^2^ lower BMI, −0.05 to −0.04) (Figure 2B, Tables S9, S10, S13). However, genetic liability to lower HbA1c was not associated with higher risk of fracture endpoints, including MOF‐site events (OR 1.02, 0.998 to 1.03), combined spine‐and‐hip fractures (OR 1.01, 0.98 to 1.03), hip fracture (OR 1.03, 0.99 to 1.06) and spine fracture (OR 0.98, 0.94 to 1.02), although it was also associated with lower eBMD (*β* −0.02 T‐score units per 1 mmol/mol lower HbA1c, −0.02 to −0.01) (Figure 3B, Tables S14–S16). The protective association of GIP‐GIPR pathway perturbation was not observed in genome‐wide liability analyses for BMI or HbA1c.

### 3.6 Time‐To‐Event Analyses and Absolute Risk Estimates Support the Primary GIP‐GIPR Findings

In time‐to‐event analyses, per SD change in GIP‐GIPR GRS corresponding to BMI‐ or HbA1c‐lowering effects, the hazard of MOF‐site fracture (HR 0.976, 0.959 to 0.992) and combined spine‐and‐hip fracture (HR 0.971, 0.946 to 0.996) was lower (Figure 5A) (Table S17). Analyses included 396 720 participants free of MOF‐site events at baseline, accruing 3.78 million person‐years of follow‐up and 13 788 incident events (incidence rates of 3.65 per 1000 person‐years). Sex‐stratified and sensitivity analyses using alternative instruments yielded directionally concordant associations.

**FIGURE 5:**
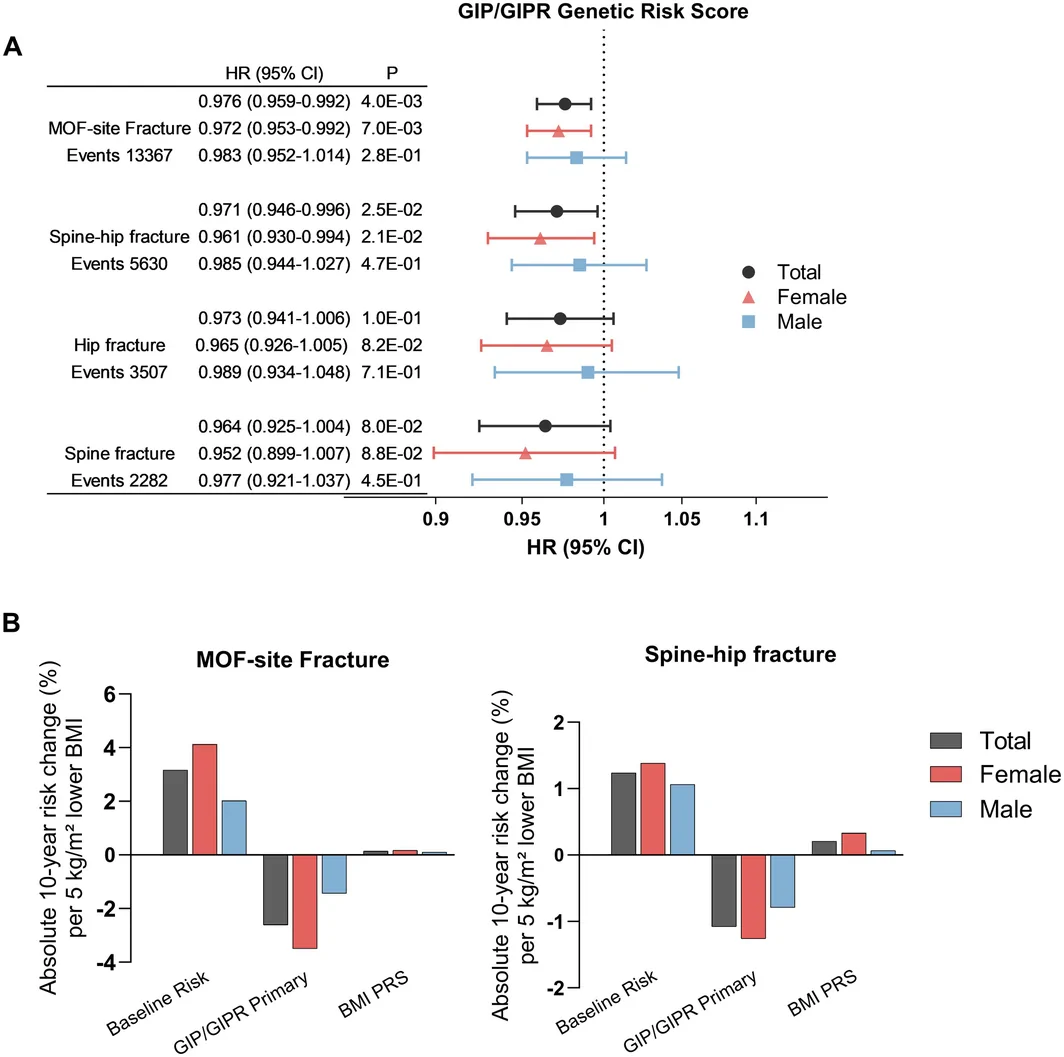
Incident fracture risk and absolute 10‐year risk reduction associated with the GIP‐GIPR GRS. (A) Cox proportional hazards analyses in UK Biobank showing hazard ratios for incident fractures at major osteoporotic sites and combined spine‐and‐hip fractures associated with the standardised GIP‐GIPR GRS. Sex‐stratified analyses are shown for the overall population (circles), females (triangles) and males (squares). (B) Absolute 10‐year risk differences for fractures at major osteoporotic sites and combined spine‐and‐hip fractures, estimated by applying Cox hazard ratios to the observed baseline cumulative incidence and scaling the genetic association estimates to a 5 kg/m^2^ lower BMI. Negative values indicate lower risk. Analyses included 386 696 individuals for the primary GIP‐GIPR score analysis and 394 914 individuals for the polygenic BMI score analysis. Error bars indicate 95% confidence intervals. BMI, body mass index; CI, confidence interval; GRS, genetic risk score; HR, hazard ratio.

When scaled to genetically predicted downstream differences corresponding to 5 kg/m^2^ lower BMI, the absolute 10‐year risk reduction was 2.62% for MOF‐site events and 1.08% for combined spine‐and‐hip fractures (Figure 5B). When scaled to 5 mmol/mol lower HbA1c, the corresponding absolute risk reductions were 2.63% and 1.08%, respectively. Findings were similar in Aalen‐Johansen competing risk sensitivity analyses (Tables S18 and S19).

For GCG‐GLP1R, estimates were imprecise across most fracture and control outcomes and should be interpreted cautiously. BMI‐weighted GCG‐GLP1R GRS was only associated with lower hazard of spine fracture (HR 0.956, 0.917 to 0.995). HbA1c‐weighted GCG‐GLP1R GRS was also associated with lower risk of spine fracture (HR 0.958, 0.920 to 0.997) and renal failure (HR 0.976, 0.961 to 0.992) (Table S17).

### 3.7 External Validation and Colocalization

External validation showed directionally concordant estimates, but estimates were often imprecise (Tables S20–S23). In HbA1c‐scaled analyses, GIP‐GIPR perturbation was associated with lower risk of osteoporosis in MVP (IVW OR 0.42, 0.23 to 0.75), with GIPR colocalization providing supportive evidence for a shared causal signal (conditional posterior probability 0.91). For GLP1R, there was no consistent effect or supportive colocalization. In BMI‐scaled analyses, GIP‐GIPR perturbation was associated with lower risk of lumbar spine and pelvis fractures (IVW OR 0.34, 0.15 to 0.79; conditional posterior probability 0.98). Cis‐regional incretin pathway perturbation showed no significant association with DXA‐measured BMD.

Sensitivity analyses using missense‐variant instruments showed associations with lower odds for some prespecified positive control outcomes and broadly consistent fracture associations with the primary GIP‐GIPR findings, although estimates were generally less precise (Tables S11 and S12). GLP1R missense analyses showed outcome‐specific associations with wider confidence intervals and should therefore be interpreted cautiously given the inconsistent evidence across positive control outcomes.

## 4 Discussion

In this human genetic study, we addressed a major safety question surrounding incretin‐based therapies by evaluating whether perturbation of incretin signalling is associated with fracture risk. We observed an association between genetically proxied GIP‐GIPR pathway perturbation and lower fracture risk, whereas evidence for GCG‐GLP1R was less consistent. These findings were supported by several complementary analyses and differed from patterns observed for genome‐wide liability to lower BMI or HbA1c.

These results provide evidence relevant to the skeletal safety of incretin‐based therapies, a question that remains unresolved despite widespread clinical use [5, 6]. Randomised trials have generally not been powered for fracture outcomes [7, 8], and observational studies have reported mixed findings but are susceptible to confounding and biases related to weight loss and healthcare utilisation [9, 10, 11]. Our findings provide complementary evidence from a human genetic framework that is less vulnerable to these biases.

The association pattern for GIP‐GIPR perturbation differed from that observed for genome‐wide genetic liability to lower BMI or HbA1c. Genetic liability to lower BMI was associated with higher fracture risk and lower bone density, consistent with established links between weight loss and skeletal fragility [38, 39]. In contrast, GIP‐GIPR perturbation was associated with lower fracture risk despite being scaled to BMI and HbA1c. This difference suggests that the observed GIP‐GIPR association does not simply recapitulate the skeletal pattern of generalised lower‐BMI liability, a finding consistent with a recent target‐trial emulation in which GLP‐1 receptor agonist initiation was associated with lower fragility‐fracture risk not explained by changes in BMI or HbA1c [40]. However, these comparisons do not establish independence from BMI or HbA1c, nor do they identify a specific biological mechanism underlying the association.

We did not observe an association between the primary GIP‐GIPR pathway‐level instruments and estimated bone mineral density captured by calcaneal ultrasound. Importantly, this finding does not allow us to determine whether GIP‐GIPR perturbation influences other determinants of fracture risk, as this study could not directly assess bone microarchitecture, muscle mass, trauma mechanism, or other non‐BMD determinants of fracture risk. Further studies incorporating imaging‐based bone phenotypes and functional outcomes will be important to clarify these mechanisms.

Evidence for the GCG‐GLP1R pathway was less consistent, with protective associations for spine fracture not uniformly supported across control outcomes and sensitivity analyses. These results suggest that inference for GLP1‐related pathways is more sensitive to instrument definition. Accordingly, these analyses were treated as exploratory.

Sex‐stratified estimates were more precise in females than males, potentially reflecting differences in fracture event counts and statistical power, and should not be interpreted as evidence of sex‐specific effect modification.

This study has several limitations. Fracture outcomes were defined using healthcare records and may include a mixture of low‐ and high‐trauma events, although such definitions capture relevant genetic susceptibility [29]. Multiple strategies were used to assess pleiotropic bias, but these cannot fully exclude horizontal pleiotropy or establish that the observed associations are mediated specifically through the GIP‐GIPR pathway, consistent with the inherent limitations of Mendelian randomisation. Genetic proxies represent lifelong pathway perturbation and may not reflect the magnitude, timing, or pharmacokinetics of therapeutic exposure, particularly given the higher potency and sustained duration of pharmacological compared with endogenous incretin signalling. For GIPR, human genetic studies have identified variants associated with lower BMI that alter or reduce receptor function, whereas pharmacological GIPR agonism produces weight loss through receptor activation. This difference in molecular mechanism emphasises that our findings cannot be directly extrapolated to fracture outcomes under GIPR‐agonist treatment and should be interpreted as reflecting pathway perturbation rather than direct pharmacological effects. Finally, the main analyses were conducted in White British participants, which may limit generalisability to other ancestral groups.

In summary, genetic evidence suggests an association between genetically proxied GIP‐GIPR pathway perturbation and lower fracture risk. These findings should not be interpreted as evidence that pharmacological GIPR agonism reduces fracture risk but support fracture‐focused evaluation of GIPR‐targeting therapies in clinical and post‐marketing studies.

## Author Contributions

**Wenjie Cheng:** conceptualization; methodology; validation; data curation; formal analysis; investigation; visualisation; writing – original draft; writing – review and editing. **Weijie Liu:** methodology; investigation; writing – review and editing. **Jie Wei:** conceptualization; methodology; supervision; writing – review and editing. **Chao Zeng:** conceptualization; methodology; supervision; writing – review and editing. **Houchen Lyu:** conceptualization; methodology; investigation; supervision; project administration; writing – original draft; writing – review and editing. **Sizheng Steven Zhao:** conceptualization; methodology; formal analysis; data curation; validation; resources; investigation; supervision; project administration; funding acquisition; writing – original draft; writing – review and editing.

## Funding

S.S.Z. is supported by an Arthritis UK Career Development Fellowship (23258), and works in centres supported by Arthritis UK (21173, 21754 and 21755) and the NIHR Manchester Biomedical Research Centre (NIHR203308).

## Ethics Statement

UK Biobank has ethical approval as a Research Tissue Bank (REC 11/NW/0382) on 17 June 2011. All participants provided informed consent. FinnGen and MVP analyses used consortium summary statistics under their respective ethics frameworks. This research was conducted under UK Biobank application 72723.

## Conflicts of Interest

S.S.Z. has received support for conference attendance from Novartis, UCB, AlfaSigma, Eli Lilly and consultancy/speaker fees from Novartis, UCB, AlfaSigma, AbbVie, Jassen, Khanda Therapeutics. The other authors declare no conflicts of interest.

## Supporting information


**Table S1:** *GIP/GIPR/GCG/GLP1R* genetic variants used to construct incretin pathway instruments.
**Table S2:** Genetic risk score construction of GIP‐GIPR and GCG‐GLP1R.
**Table S3:** ICD‐10 codes used to identify outcomes.
**Table S4:** Results of GRS by sex interaction analyses for outcome traits.
**Table S5:** Sample sizes of the outcome GWAS datasets used in the two‐sample MR analysis.
**Table S6:** Missense instruments for sensitivity analyses.
**Table S7:** Baseline characteristics of participants with complete BMI and HbA1c data.
**Table S8:** Associations of genetic risk scores (GRS) with control outcomes.
**Table S9:** Associations of GRS with fracture outcomes in the overall population, scaled to 1 kg/m^2^ lower BMI.
**Table S10:** Sex‐stratified associations of GRS with fracture outcomes, scaled to 1 kg/m^2^ lower BMI.
**Table S11:** Associations of individual GIP‐GIPR and missense variants with fracture and control outcomes in the overall and sex‐stratified populations.
**Table S12:** Associations of individual GIP‐GIPR and missense variants with estimated bone mineral density (eBMD) in the overall and sex‐stratified populations.
**Table S13:** Associations of GRS with estimated bone mineral density (eBMD) in the overall and sex‐stratified populations, scaled to 1 kg/m^2^ lower BMI.
**Table S14:** Associations of GRS with fracture outcomes in the overall population, scaled to 1 mmol/mol lower HbA1c.
**Table S15:** Sex‐stratified associations of GRS with fracture outcomes, scaled to 1 mmol/mol lower HbA1c.
**Table S16:** Associations of GRS with estimated bone mineral density (eBMD) in the overall and sex‐stratified populations, scaled to 1 mmol/mol lower HbA1c.
**Table S17:** Associations of GRS with fracture outcomes using Cox proportional hazards models, scaled to lower BMI or HbA1c.
**Table S18:** Kaplan–Meier estimates of fracture risk according to incretin pathway genetic risk scores (GRS), scaled to 5 kg/m^2^ lower BMI or 5 mmol/mol lower HbA1c.
**Table S19:** Aalen–Johansen cumulative incidence of fracture outcomes according to incretin pathway genetic risk scores (GRS), scaled to 5 kg/m^2^ lower BMI or 5 mmol/mol lower HbA1c.
**Table S20:** Two‐sample Mendelian randomisation estimates for fracture and osteoporosis outcomes.
**Table S21:** Two‐sample Mendelian randomisation estimates for BMD.
**Table S22:** MR‐Egger intercept tests for directional pleiotropy.
**Table S23:** Colocalization analyses.

**Data S1:** STROBE‐MR checklist of recommended items to address in reports of Mendelian randomisation studies.

## Data Availability

Data from UK Biobank are available to bona fide researchers for health‐related research in the public interest, subject to registration, application and approval by UK Biobank. FinnGen and Million Veteran Program summary statistics are available through their respective data access procedures. Other GWAS summary statistics used in this study are publicly available from the cited sources. All analytical outputs generated in the present study are presented in the Supporting Informations.
