# Supplementary material for: Genetically Proxied GIP‐GIPR Pathway Perturbation and Fracture Risk
Source: Diabetes Obes Metab. 2026 Sep 29;28(12):11839–50. doi: 10.1111/dom.71378 (PMC13731033; doi:10.1111/dom.71378)
Supplement: Supplementary file 1 [file DOM-28-11839-s002.docx]

**STROBE-MR checklist of recommended items to address in reports of Mendelian randomization studies**^1^ ^2^

| **Item No.** | **Section** | **Checklist item** | **Page No.** | **Relevant text from manuscript** |
| --- | --- | --- | --- | --- |
| 1 | **TITLE and ABSTRACT** | Indicate Mendelian randomization (MR) as the study’s design in the title and/or the abstract if that is a main purpose of the study | 3 | We conducted drug-target Mendelian randomisation using cis genetic variants near GIP, GIPR, GCG, GLP1R in 401,983 unrelated UK Biobank participants. Instruments were scaled to 1 kg/m² lower BMI or 1 mmol/mol lower HbA1c. Outcomes included fractures at major osteoporotic sites, combined spine-and-hip fractures, site-specific fractures, and estimated bone mineral density (eBMD). We compared these estimates with genome-wide liability to lower BMI or HbA1c to contextualise the fracture associations observed for the target-proximal instruments. We used Cox models to estimate time-to-fracture associations per SD of target genetic risk score aligned to lower BMI or HbA1c. |
|  | **INTRODUCTION** |  |  |  |
| 2 | **Background** | Explain the scientific background and rationale for the reported study. What is the exposure? Is a potential causal relationship between exposure and outcome plausible? Justify why MR is a helpful method to address the study question | 5 | “Incretin-based therapies are rapidly reshaping the management of type 2 diabetes and obesity, exposing millions of individuals to sustained GLP-1 receptor and, increasingly, GIP/GLP-1 receptor activation (1, 2). As these agents expand into broader metabolic disease populations, their safety has become increasingly important (3, 4). One clinically relevant but unresolved question is whether long-term perturbation of incretin signalling influences skeletal health and fracture risk.  Existing evidence has not resolved whether incretin therapies increase, decrease, or have no meaningful effect on fracture risk (5, 6). Randomised trials have generally not been designed or powered to assess fracture outcomes (7, 8), and observational studies can be challenging to interpret because users differ in characteristics that are also important for fracture risk (9-11). These limitations are particularly relevant for newer agents targeting the glucose-dependent insulinotropic polypeptide (GIP) receptor and for dual GIPR/GLP-1 receptor (GLP1R) agonists, where clinical use is expanding but long-term skeletal safety data are sparse. Mechanistically, the direction of effect is uncertain: improvements in glycaemic control and reductions in inflammation could plausibly benefit skeletal health (6, 12), whereas loss of lean mass may increase susceptibility to falls and fractures (13, 14).  Human genetics offer a complementary way to address this question (15). Genetic variations in genes encoding incretin pathway drug targets can proxy their lifelong perturbation and enable assessment of fracture outcomes with less susceptibility to confounding by indication than conventional observational studies (16).” |
| 3 | **Objectives** | State specific objectives clearly, including pre-specified causal hypotheses (if any). State that MR is a method that, under specific assumptions, intends to estimate causal effects | 5 | “We therefore examined whether genetically proxied perturbation of incretin signalling—focusing on the GIPR and GLP1R pathways—was associated with fracture risk and bone mineral density.” |
|  | **METHODS** |  |  |  |
| 4 | **Study design and data sources** | Present key elements of the study design early in the article. Consider including a table listing sources of data for all phases of the study. For each data source contributing to the analysis, describe the following: | 6-11 |  |
|  | a) | Setting: Describe the study design and the underlying population, if possible. Describe the setting, locations, and relevant dates, including periods of recruitment, exposure, follow-up, and data collection, when available. | 6 | **Study overview**  We conducted a drug-target Mendelian randomisation (MR) study using genetic variants in or near the genes encoding each drug target (combined into a genetic risk score, GRS) to proxy perturbation of incretin signalling. Focusing on downstream biomarkers (BMI and HbA1c), we tested associations with fracture outcomes and estimated heel bone mineral density (eBMD), and performed complementary GRS-based time-to-event analyses using individual-level UK Biobank data. Where possible, we attempted validation using two-sample MR with summary statistics from FinnGen (17) (freeze 12) and the Million Veteran Program (18) (MVP). |
|  | b) | Participants: Give the eligibility criteria, and the sources and methods of selection of participants. Report the sample size, and whether any power or sample size calculations were carried out prior to the main analysis | 6 | **UK Biobank Participants**  UK Biobank recruited approximately 500,000 individuals aged 40-69 years between 2006 and 2010, with linked health outcome data (19). We restricted analyses to unrelated participants (kinship coefficient <0.0884) of White British ancestry with concordant genetic and self-reported sex. The final analytic sample included 401,983 individuals. Each analysis included the largest subset of participants with complete data for the relevant exposure and outcome. |
|  | c) | Describe measurement, quality control and selection of genetic variants | 6-8 | **Genotyping, imputation and genetic quality control**  Genotyping was performed centrally by UK Biobank using the UK BiLEVE Axiom array for the initial subset and the UK Biobank Axiom array for the remainder, with standard sample- and variant-level quality control conducted by UK Biobank (19). UK Biobank phased and imputed genotypes using reference panels including the Haplotype Reference Consortium (HRC) and a merged UK10K/1000 Genomes reference where required. In this study, we restricted variants to those with imputation INFO score > 0.9 and minor allele frequency > 1%.  **Genetic instruments for incretin signalling**  Cis genetic instruments were constructed using variants within 250 kb of genes encoding incretin ligands and receptors, including glucose-dependent insulinotropic polypeptide (GIP), its receptor (GIPR), proglucagon (GCG), and the glucagon-like peptide-1 receptor (GLP1R). GLP1 is a cleavage product of proglucagon encoded by the GCG gene, thus variants near GCG were used to proxy upstream GLP1 ligand biology. Variants were selected from external European-ancestry GWAS of BMI(20) or HbA1c(21), prioritising datasets without UK Biobank overlap to minimize winner’s curse. Independent variants (r² < 0.1)(22) reaching genome-wide significance were used where available; otherwise regional significant (P ≤ 5×10⁻⁶) variants were used if instrument strength was adequate based on F-statistics. Regional significance threshold is widely adopted in cis-MR. (23-25) Variants with opposing effects on BMI and HbA1c (P < 0.05) were excluded to avoid combining variants with discordant downstream metabolic associations and to preserve a consistent phenotypic orientation of the genetic instrument.  Primary instruments were constructed for two pathways: GIP-GIPR and GCG-GLP1R. For GIP-GIPR, two variants (rs35895680 and rs2302593) were selected for the primary instrument. Given the small number of variants, each was also analysed separately to assess the contribution of the individual variants to the observed associations. Additional variants, including missense variants, were evaluated. For GCG-GLP1R, no variants met the prespecified criteria in external datasets; selection was therefore extended to GWAS including UK Biobank data using a relaxed threshold (P ≤ 5×10⁻⁵)(26, 27). Detailed characteristics of the selected variants, including the source datasets, discovery P-values, and UK Biobank association statistics, are provided in Supplementary Table S1.  GRS were constructed by summing effect alleles. Alleles were aligned according to their downstream phenotypic association, such that the effect allele corresponded to lower BMI or lower HbA1c, and genotypes were coded as 0, 1 or 2 copies and summed to generate each score. Unweighted GRS were used for the primary instruments. Conditional weighting was applied only for the GIP-GIPR sensitivity score containing variants in modest LD (r² = 0.185), using adjusted β coefficients from linear regression of the exposure on each variant, adjusting for the remaining variants in the score. Instrument strength was assessed using the F statistic,(28) with F > 10 indicating adequate strength. Details of GRS construction are provided in Supplementary Table S2.  Body mass index (BMI, kg/m²) was calculated from measured height and weight at baseline. Glycated haemoglobin (HbA1c, mmol/mol) was measured using high-performance liquid chromatography (VARIANT II Turbo, Bio-Rad). Implausible values were excluded (BMI <10 or >60 kg/m²; HbA1c outside 15-184 mmol/mol). Genetic effects were scaled to BMI or HbA1c to provide clinically interpretable measures of the genetically proxied pathway perturbation. |
|  | d) | For each exposure, outcome, and other relevant variables, describe methods of assessment and diagnostic criteria for diseases | 8 | Body mass index (BMI, kg/m²) was calculated from measured height and weight at baseline. Glycated haemoglobin (HbA1c, mmol/mol) was measured using high-performance liquid chromatography (VARIANT II Turbo, Bio-Rad). Implausible values were excluded (BMI <10 or >60 kg/m²; HbA1c outside 15-184 mmol/mol). Genetic effects were scaled to BMI or HbA1c to provide clinically interpretable measures of the genetically proxied pathway perturbation.  **Outcomes**  Fracture outcomes were derived from linked hospital admission data and mortality records using International Classification of Diseases, 10th revision (ICD-10) codes(29-31) (Supplementary Table S3). The primary outcome was a site-based major osteoporotic fracture phenotype (MOF-site), defined as fractures at the thoracic or lumbar spine, hip/femur, pelvis, humerus, or forearm, together with selected vertebral collapse, fatigue fracture codes and osteoporosis-with-pathological-fracture codes at corresponding sites. This phenotype was not intended to distinguish fractures by trauma mechanism and therefore may include both low- and high-trauma fractures. Secondary outcomes included combined spine-and-hip fracture, isolated spine fracture and isolated hip fracture. The combined spine and-hip fractures were defined to increase statistical power by enlarging the case sample size, and these secondary outcomes are partially overlapping. Estimated bone mineral density (eBMD) was measured by calcaneal quantitative ultrasound in approximately half of participants at baseline and reported as sex-matched T-scores, representing bone density deviations from sex-specific reference values in standard deviation (SD) units. |
|  | e) | Provide details of ethics committee approval and participant informed consent, if relevant | 22 | **Ethics**  UK Biobank has ethical approval as a Research Tissue Bank (REC 11/NW/0382). All participants provided informed consent. This research was conducted under UK Biobank application 72723. FinnGen and MVP analyses used consortium summary statistics under their respective ethics frameworks. |
| 5 | **Assumptions** | Explicitly state the three core IV assumptions for the main analysis (relevance, independence and exclusion restriction) as well assumptions for any additional or sensitivity analysis | 9-10 | **Control outcomes**  As a prespecified assessment of instrument performance, we examined positive control outcomes (heart failure and chronic renal failure) based on established effects of incretin therapies,(3, 32, 33) and a negative control outcome (ankylosing spondylitis) with no known mechanistic link to incretin signalling. The prespecified interpretive hierarchy required an exposure F-statistic > 10, directionally concordant associations with both positive controls reaching nominal significance (P < 0.05), and no statistical evidence of association with the negative control (P > 0.05) for an instrument to be prioritised for primary interpretation. Instruments not meeting these criteria were reported in full but interpreted as exploratory. These control analyses were intended to assess consistency with the expected downstream phenotype rather than to establish pharmacological equivalence or exclude pleiotropy. |
| 6 | **Statistical methods: main analysis** | Describe statistical methods and statistics used | 9 |  |
|  | a) | Describe how quantitative variables were handled in the analyses (i.e., scale, units, model) | 10 | Estimated bone mineral density (eBMD) was measured by calcaneal quantitative ultrasound in approximately half of participants at baseline and reported as sex-matched T-scores, representing bone density deviations from sex-specific reference values in standard deviation (SD) units.  Body mass index (BMI, kg/m²) was calculated from measured height and weight at baseline. Glycated haemoglobin (HbA1c, mmol/mol) was measured using high-performance liquid chromatography (VARIANT II Turbo, Bio-Rad). Implausible values were excluded (BMI <10 or >60 kg/m²; HbA1c outside 15-184 mmol/mol). Genetic effects were scaled to BMI or HbA1c to provide clinically interpretable measures of the genetically proxied pathway perturbation. |
|  | b) | Describe how genetic variants were handled in the analyses and, if applicable, how their weights were selected | 8-9 | **Genetic instruments for incretin signalling**  Cis genetic instruments were constructed using variants within 250 kb of genes encoding incretin ligands and receptors, including glucose-dependent insulinotropic polypeptide (GIP), its receptor (GIPR), proglucagon (GCG), and the glucagon-like peptide-1 receptor (GLP1R). GLP1 is a cleavage product of proglucagon encoded by the GCG gene, thus variants near GCG were used to proxy upstream GLP1 ligand biology. Variants were selected from external European-ancestry GWAS of BMI(20) or HbA1c(21), prioritising datasets without UK Biobank overlap to minimize winner’s curse. Independent variants (r² < 0.1)(22) reaching genome-wide significance were used where available; otherwise regional significant (P ≤ 5×10⁻⁶) variants were used if instrument strength was adequate based on F-statistics. Regional significance threshold is widely adopted in cis-MR. (23-25) Variants with opposing effects on BMI and HbA1c (P < 0.05) were excluded to avoid combining variants with discordant downstream metabolic associations and to preserve a consistent phenotypic orientation of the genetic instrument.  Primary instruments were constructed for two pathways: GIP-GIPR and GCG-GLP1R. For GIP-GIPR, two variants (rs35895680 and rs2302593) were selected for the primary instrument. Given the small number of variants, each was also analysed separately to assess the contribution of the individual variants to the observed associations. Additional variants, including missense variants, were evaluated. For GCG-GLP1R, no variants met the prespecified criteria in external datasets; selection was therefore extended to GWAS including UK Biobank data using a relaxed threshold (P ≤ 5×10⁻⁵)(26, 27). Detailed characteristics of the selected variants, including the source datasets, discovery P-values, and UK Biobank association statistics, are provided in Supplementary Table S1.  GRS were constructed by summing effect alleles. Alleles were aligned according to their downstream phenotypic association, such that the effect allele corresponded to lower BMI or lower HbA1c, and genotypes were coded as 0, 1 or 2 copies and summed to generate each score. Unweighted GRS were used for the primary instruments. Conditional weighting was applied only for the GIP-GIPR sensitivity score containing variants in modest LD (r² = 0.185), using adjusted β coefficients from linear regression of the exposure on each variant, adjusting for the remaining variants in the score. Instrument strength was assessed using the F statistic,(28) with F > 10 indicating adequate strength. Details of GRS construction are provided in Supplementary Table S2.  Body mass index (BMI, kg/m²) was calculated from measured height and weight at baseline. Glycated haemoglobin (HbA1c, mmol/mol) was measured using high-performance liquid chromatography (VARIANT II Turbo, Bio-Rad). Implausible values were excluded (BMI <10 or >60 kg/m²; HbA1c outside 15-184 mmol/mol). Genetic effects were scaled to BMI or HbA1c to provide clinically interpretable measures of the genetically proxied pathway perturbation. |
|  | c) | Describe the MR estimator (e.g. two-stage least squares, Wald ratio) and related statistics. Detail the included covariates and, in case of two-sample MR, whether the same covariate set was used for adjustment in the two samples | 9-11 | **Statistical analysis**  **One-sample Mendelian randomization**  All analyses were conducted in R (version 4.5.1). One-sample MR used the ratio approach. The unweighted GRS was treated as a single score-level genetic instrument. For each GRS, associations with binary outcomes (fractures and controls) were estimated using logistic regression and associations with eBMD were estimated using linear regression. The corresponding GRS-exposure association was estimated using linear regression. Models were adjusted for age, sex (where applicable) and PC1-PC10. The ratio estimate was computed as β(GRS→outcome)/β(GRS→exposure). For binary outcomes, the logistic-regression coefficient on the log-odds scale was divided by the GRS-exposure coefficient; the resulting ratio estimate was exponentiated and reported as an odds ratio per 1 kg/m² lower BMI or per 1 mmol/mol lower HbA1c. Standard errors were obtained using the delta method.  Sex-stratified analyses were performed as descriptive analyses. GRS-by-sex interaction terms were included in regression models to formally assess sex-dependent effects, with interaction P-values reported in Supplementary Table S4.  **Time-to-event analyses**  Cox proportional hazards models were used to estimate associations with incident fracture outcomes. Participants entered the risk set at their UK Biobank baseline assessment date. Individuals with a relevant fracture code before baseline were excluded from the corresponding incident outcome analyses. Follow-up commenced at baseline and continued until the first fracture event, death, the last valid follow-up observation, or the administrative end of linked Hospital Episode Statistics (HES) data (April 2022), whichever occurred first. Models were adjusted for age, sex, and PC1-PC10, with analyses repeated in sex-stratified models. Each GRS was standardized to z-scores.  Absolute risk differences at 10 years corresponding to a 5 kg/m² reduction in BMI or a 5 mmol/mol reduction in HbA1c were estimated by applying hazard ratios to observed baseline cumulative incidence. To account for competing risks and avoid bias from non-informative censoring, we performed complementary analyses using the Aalen-Johansen (AJ) model.  **External validation and sensitivity analyses**  Two-sample MR analyses were conducted using fracture outcome summary statistics from FinnGen(17) and MVP(18), with DXA-measured BMD(34) expressed as SD units (g/cm²) additionally included in analyses (Supplementary Table S5). Fixed-effect inverse-variance weighted models were used as the primary approach, with MR-Egger as a sensitivity analysis. Directional pleiotropy was assessed using the MR-Egger intercept. Multiple testing was addressed using false discovery rate (FDR) correction across outcomes for each instrument.  Genetic instruments were derived from BMI(26) and HbA1c(27) GWAS. Variants within ±250 kb of the GIP-GIPR and GCG-GLP1R loci reaching significance (P < 5×10⁻⁸) were selected as cis instruments and pruned for linkage disequilibrium (r² < 0.1). Instruments prioritized ligand-receptor perturbation where possible (e.g., GIP-GIPR). Genome-wide BMI- and HbA1c-associated variants outside these regions were selected at P < 5×10⁻⁸ and pruned using r² < 0.001.  Colocalization analyses were performed for associations with IVW P < 0.05 within ±50 kb of the cis-region using default priors (p1 = 1×10⁻⁴, p2 = 1×10⁻⁴, p12 = 1×10⁻⁵), with strong evidence defined as P4/(P3+P4) > 0.8. Analyses were conducted in R using the TwoSampleMR(35), MendelianRandomization(36), and coloc(37) packages.  Sensitivity analyses also included missense variants used in published studies, some of which were not included in the primary instruments, for example, because of a minor allele frequency <1% (24, 25, 32) (Supplementary Table S6). Genome-wide polygenic risk scores (PRS) for BMI and HbA1c were additionally included as contextual comparators, derived from external GWAS and calculated in UK Biobank participants. |
|  | d) | Explain how missing data were addressed | 6 | Each analysis included the largest subset of participants with complete data for the relevant exposure and outcome. |
|  | e) | If applicable, indicate how multiple testing was addressed | 10 | Multiple testing was addressed using false discovery rate (FDR) correction across outcomes for each instrument. |
| 7 | **Assessment of assumptions** | Describe any methods or prior knowledge used to assess the assumptions or justify their validity | 9 | **Control outcomes**  As a prespecified assessment of instrument performance, we examined positive control outcomes (heart failure and chronic renal failure) based on established effects of incretin therapies,(3, 32, 33) and a negative control outcome (ankylosing spondylitis) with no known mechanistic link to incretin signalling. The prespecified interpretive hierarchy required an exposure F-statistic > 10, directionally concordant associations with both positive controls reaching nominal significance (P < 0.05), and no statistical evidence of association with the negative control (P > 0.05) for an instrument to be prioritised for primary interpretation. Instruments not meeting these criteria were reported in full but interpreted as exploratory. These control analyses were intended to assess consistency with the expected downstream phenotype rather than to establish pharmacological equivalence or exclude pleiotropy. |
| 8 | **Sensitivity analyses and additional analyses** | Describe any sensitivity analyses or additional analyses performed (e.g. comparison of effect estimates from different approaches, independent replication, bias analytic techniques, validation of instruments, simulations) | 9-10 | External validation and sensitivity analyses  Two-sample MR analyses were conducted using fracture outcome summary statistics from FinnGen(17) and MVP(18), with DXA-measured BMD(34) expressed as SD units (g/cm²) additionally included in analyses (Supplementary Table S5). Fixed-effect inverse-variance weighted models were used as the primary approach, with MR-Egger as a sensitivity analysis. Directional pleiotropy was assessed using the MR-Egger intercept. Multiple testing was addressed using false discovery rate (FDR) correction across outcomes for each instrument.  Genetic instruments were derived from BMI(26) and HbA1c(27) GWAS. Variants within ±250 kb of the GIP-GIPR and GCG-GLP1R loci reaching significance (P < 5×10⁻⁸) were selected as cis instruments and pruned for linkage disequilibrium (r² < 0.1). Instruments prioritized ligand-receptor perturbation where possible (e.g., GIP-GIPR). Genome-wide BMI- and HbA1c-associated variants outside these regions were selected at P < 5×10⁻⁸ and pruned using r² < 0.001.  Colocalization analyses were performed for associations with IVW P < 0.05 within ±50 kb of the cis-region using default priors (p1 = 1×10⁻⁴, p2 = 1×10⁻⁴, p12 = 1×10⁻⁵), with strong evidence defined as P4/(P3+P4) > 0.8. Analyses were conducted in R using the TwoSampleMR(35), MendelianRandomization(36), and coloc(37) packages.  Sensitivity analyses also included missense variants used in published studies, some of which were not included in the primary instruments, for example, because of a minor allele frequency <1% (24, 25, 32) (Supplementary Table S6). Genome-wide polygenic risk scores (PRS) for BMI and HbA1c were additionally included as contextual comparators, derived from external GWAS and calculated in UK Biobank participants. |
| 9 | **Software and pre-registration** |  |  |  |
|  | a) | Name statistical software and package(s), including version and settings used | 10 | “All analyses were conducted in R (version 4.5.1).”  “All analyses were conducted in R using the TwoSampleMR, MendelianRandomization, and coloc packages.” |
|  | b) | State whether the study protocol and details were pre-registered (as well as when and where) | 10 | “This research was conducted under UK Biobank application 72723.” |
|  | **RESULTS** |  |  |  |
| 10 | **Descriptive data** |  |  |  |
|  | a) | Report the numbers of individuals at each stage of included studies and reasons for exclusion. Consider use of a flow diagram | 12  15 | Analyses included 401,983 unrelated White British participants from the UK Biobank (Supplementary Table S7).  These analyses included 396720 participants free of MOF-site events at baseline followed for 3.78 million person-years, during which 13,788 incident MOF-site events occurred (incidence rates of 3.65 per 1,000 person years). |
|  | b) | Report summary statistics for phenotypic exposure(s), outcome(s), and other relevant variables (e.g. means, SDs, proportions) | Supplementary Table S7 | ST7. Baseline characteristics of participants with complete BMI and HbA1c data |
|  | c) | If the data sources include meta-analyses of previous studies, provide the assessments of heterogeneity across these studies | / | / |
|  | d) | For two-sample MR:  i.  Provide justification of the similarity of the genetic variant-exposure associations between the exposure and outcome samples  ii.  Provide information on the number of individuals who overlap between the exposure and outcome studies | 20-23 | **External validation and colocalization**  External validation showed directionally concordant estimates, but estimates were often imprecise (Supplementary Tables S20-23). In HbA1c-scaled analyses, GIP-GIPR perturbation was associated with lower risk of osteoporosis in MVP (IVW OR 0.42, 0.23 to 0.75), with GIPR colocalization providing supportive evidence for a shared causal signal (conditional posterior probability 0.91). For GLP1R, there was no consistent effect or supportive colocalization.In BMI-scaled analyses, GIP-GIPR perturbation was associated with lower risk of lumbar spine and pelvis fractures (IVW OR 0.34, 0.15 to 0.79; conditional posterior probability 0.98). Cis-regional incretin pathway perturbation showed no significant association with DXA-measured BMD. |
| 11 | **Main results** |  |  |  |
|  | a) | Report the associations between genetic variant and exposure, and between genetic variant and outcome, preferably on an interpretable scale | Supplementary Table S2 | ST2. Genetic Risk Score Construction of GIP-GIPR and GCG-GLP1R |
|  | b) | Report MR estimates of the relationship between exposure and outcome, and the measures of uncertainty from the MR analysis, on an interpretable scale, such as odds ratio or relative risk per SD difference | 11-16 | “Genetic perturbation of the GIP-GIPR pathway scaled to 1 kg/m² lower BMI was associated with lower risk of ICD-coded fractures at major osteoporotic sites (MOF-site events) (OR 0.80, 0.66 to 0.98) (Fig.2A). Protective associations were also observed for secondary fracture outcomes, including combined spine-and-hip fractures (OR 0.68, 0.49 to 0.94) and spine fractures (OR 0.60, 0.36 to 0.99), whereas estimates for hip fractures were directionally similar but imprecise (OR 0.72, 0.48 to 1.08) (Supplementary Table S9). These secondary outcomes were interpreted as supportive findings rather than independent confirmatory endpoints..”  “Genetic perturbation of the GIP-GIPR pathway scaled to 1 mmol/mol lower HbA1c was associated with lower risk of MOF-site events (OR 0.80, 0.65 to 0.98) (Fig.3A). Protective associations were also observed for combined spine-and-hip fractures (OR 0.68, 0.49 to 0.94) and spine fracture (OR 0.57, 0.35 to 0.95). Estimates for hip fractures were directionally similar but imprecise (OR 0.75, 0.50 to 1.13) (Supplementary Table S14).”  “Genetically proxied perturbation of the GCG-GLP1R pathway showed less consistent associations across fracture outcomes. HbA1c-scaled analyses suggested lower risk for some endpoints, particularly spine fracture (OR 0.61, 0.40 to 0.91), but estimates for MOF-site events (OR 0.85, 0.73 to 1.01), combined spine-and-hip fractures (OR 0.82, 0.63 to 1.06) and hip fractures (OR 0.99, 0.71 to 1.37) were less precise and not consistently supported across control outcomes and sensitivity analyses (Fig.4).”  “By contrast, genetic liability to lower BMI showed strong associations with higher fracture risk, including MOF-site events (OR 1.01, 1.0008 to 1.02), combined spine-and-hip fractures (OR 1.03, 1.01 to 1.05) and hip fractures (OR 1.05, 1.02 to 1.07), and lower eBMD (β -0.04 T-score units per 1 kg/m² lower BMI, -0.045 to -0.037) (Fig.2B, Supplementary Tables S9, 10, 13). However, genetic liability to lower HbA1c was not associated with higher risk of fracture endpoints, including MOF-site events (OR 1.02, 0.998 to 1.03), combined spine-and-hip fractures (OR 1.01, 0.98 to 1.03), hip fracture (OR 1.03, 0.99 to 1.06) and spine fracture (OR 0.98, 0.94 to 1.02), although it was also associated with lower eBMD (β -0.02 T-score units per 1 mmol/mol lower HbA1c, -0.02 to -0.01) (Fig.3B, Supplementary Tables S14-16). The protective association of GIP-GIPR pathway perturbation was not observed in genome-wide liability analyses for BMI or HbA1c..  BMI-scaled analyses were generally imprecise, although a protective association was observed for spine fracture (OR 0.45, 0.21 to 0.99) (table S3). Overall, these findings indicate more limited and less robust evidence for a role of the GLP1 pathway in fracture risk compared with GIP-GIPR (tables S3 to S8).”  “In time-to-event analyses, per SD change in GIP-GIPR GRS corresponding to BMI- or HbA1c-lowering effects, the hazard of MOF-site fracture (HR 0.976, 0.959 to 0.992) and combined spine-and-hip fracture (HR 0.971, 0.946 to 0.996) was lower (Fig.5A). These analyses included 396720 participants free of MOF-site events at baseline followed for 3.78 million person-years, during which 13,788 incident MOF-site events occurred (incidence rates of 3.65 per 1,000 person years).”  “External validation showed directionally concordant estimates, but estimates were often imprecise (Supplementary Tables S20-23). In HbA1c-scaled analyses, GIP-GIPR perturbation was associated with lower risk of osteoporosis in MVP (IVW OR 0.42, 0.23 to 0.75), with GIPR colocalization providing supportive evidence for a shared causal signal (conditional posterior probability 0.91). For GLP1R, there was no consistent effect or supportive colocalization.In BMI-scaled analyses, GIP-GIPR perturbation was associated with lower risk of lumbar spine and pelvis fractures (IVW OR 0.34, 0.15 to 0.79; conditional posterior probability 0.98). Cis-regional incretin pathway perturbation showed no significant association with DXA-measured BMD.” |
|  | c) | If relevant, consider translating estimates of relative risk into absolute risk for a meaningful time period | 15 | When scaled to genetically predicted downstream differences corresponding to 5 kg/m² lower BMI, the absolute 10-year risk reduction was 2.62% for MOF-site events and 1.08% for combined spine-and-hip fractures (Fig.5B). Corresponding estimates scaled to 5 mmol/mol lower HbA1c, the corresponding absolute risk reduction was 2.63% and 1.08% respectively. Findings were similar in Aalen-Johansen competing risk sensitivity analyses (Supplementary Tables S17-19). |
|  | d) | Consider plots to visualize results (e.g. forest plot, scatterplot of associations between genetic variants and outcome versus between genetic variants and exposure) | 27-30 | Fig2-5 |
| 12 | **Assessment of assumptions** |  |  |  |
|  | a) | Report the assessment of the validity of the assumptions | 11 | **Associations of incretin pathway instruments with control outcomes**  Analyses included 401,983 unrelated White British participants from the UK Biobank (Supplementary Table S5).  The GIP-GIPR instrument showed the strongest and most consistent evidence across positive control outcomes. The HbA1c-scaled GCG-GLP1R instrument showed partial concordance with positive controls, whereas the BMI-scaled GCG-GLP1R instrument was interpreted cautiously because of inconsistent signals across control outcomes. Instrument characteristics are summarized in Supplementary Tables S2.  Genetically proxied GIP-GIPR perturbation showed protective associations with both positive controls when scaled to BMI (heart failure OR 0.67, 95% CI 0.52 to 0.86; chronic renal failure OR 0.73, 0.58 to 0.92) and when scaled to HbA1c (heart failure OR 0.68, 0.53 to 0.87; chronic renal failure OR 0.74, 0.59 to 0.93), with no evidence of association with the negative control (OR 1.33, 0.56 to 3.17 for BMI scaling; OR 1.42, 0.61 to 3.3 for HbA1c scaling).  Genetically proxied GCG-GLP1R perturbation showed concordant associations across positive controls: HbA1c-scaled estimates indicated lower risk of chronic renal failure (OR 0.76, 0.64 to 0.92), whereas estimates for heart failure lacked precision (OR 0.90, 0.74 to 1.09). For BMI-scaled GCG-GLP1R perturbation, confidence intervals were wide across all control outcomes (Supplementary Table S6). |
|  | b) | Report any additional statistics (e.g., assessments of heterogeneity across genetic variants, such as *I^2^*, Q statistic or E-value) | 15 | “Directional pleiotropy was assessed using the MR-Egger intercep.” |
| 13 | **Sensitivity analyses and additional analyses** |  |  |  |
|  | a) | Report any sensitivity analyses to assess the robustness of the main results to violations of the assumptions | 15-16 | External validation and colocalization  External validation showed directionally concordant estimates, but estimates were often imprecise (Supplementary Tables S20-23). In HbA1c-scaled analyses, GIP-GIPR perturbation was associated with lower risk of osteoporosis in MVP (IVW OR 0.42, 0.23 to 0.75), with GIPR colocalization providing supportive evidence for a shared causal signal (conditional posterior probability 0.91). For GLP1R, there was no consistent effect or supportive colocalization.In BMI-scaled analyses, GIP-GIPR perturbation was associated with lower risk of lumbar spine and pelvis fractures (IVW OR 0.34, 0.15 to 0.79; conditional posterior probability 0.98). Cis-regional incretin pathway perturbation showed no significant association with DXA-measured BMD.  Sensitivity analyses using missense-variant instruments s showed associations with lower odds for some prespecified positive control outcomes and broadly consistent fracture associations with the primary GIP-GIPR findings, although estimates were generally less precise (Supplementary Tables S11-12). GLP1R missense analyses showed outcome-specific associations with wider confidence intervals and should therefore be interpreted cautiously given the inconsistent evidence across positive control outcomes, consistent with the less consistent evidence from the primary GCG-GLP1R analyses |
|  | b) | Report results from other sensitivity analyses or additional analyses | 11-16 | “Sex-stratified analyses yielded more precise estimates in females than in males. Among females, the BMI-scaled GIP-GIPR perturbation showed directionally consistent estimates for combined spine-and-hip fractures (OR 0.65, 0.44 to 0.96) and MOF-site events (OR 0.81, 0.65 to 1.01), and estimates in males were directionally similar (Supplementary Table S10). In single-variant analyses, the GIP variant was nominally associated with lower risk of the primary MOF-site outcome and with the positive control outcomes, whereas the GIPR variant produced directionally concordant but less precise estimates. Alternative GIP-GIPR instrument specifications also showed broadly concordant associations with fracture outcomes (Supplementary Tables S11–S12)”  “Effect estimates were more precise in females. HbA1c-scaled GIP-GIPR perturbation was associated with lower odds of MOF-site events (OR 0.77, 0.61 to 0.99) and combined spine-and-hip fractures in females (OR 0.62, 0.40 to 0.95) (Supplementary Table S15). Sensitivity analyses using alternative GIP-GIPR instrument specifications yielded directionally concordant estimates across fracture outcomes, with the association for combined spine-and-hip fractures reaching nominal statistical significance (OR 0.73, 0.55 to 0.97).”  “Sex-stratified models yielded directionally concordant estimates (Supplementary Table S17). Sensitivity analyses using alternative GIP-GIPR instruments yielded directionally concordant associations for fracture outcomes.”  “External validation showed directionally concordant estimates, but estimates were often imprecise (Supplementary Tables S20-23). In HbA1c-scaled analyses, GIP-GIPR perturbation was associated with lower risk of osteoporosis in MVP (IVW OR 0.42, 0.23 to 0.75), with GIPR colocalization providing supportive evidence for a shared causal signal (conditional posterior probability 0.91). For GLP1R, there was no consistent effect or supportive colocalization.In BMI-scaled analyses, GIP-GIPR perturbation was associated with lower risk of lumbar spine and pelvis fractures (IVW OR 0.34, 0.15 to 0.79; conditional posterior probability 0.98). Cis-regional incretin pathway perturbation showed no significant association with DXA-measured BMD.  Sensitivity analyses using missense-variant instruments s showed associations with lower odds for some prespecified positive control outcomes and broadly consistent fracture associations with the primary GIP-GIPR findings, although estimates were generally less precise (Supplementary Tables S11-12). GLP1R missense analyses showed outcome-specific associations with wider confidence intervals and should therefore be interpreted cautiously given the inconsistent evidence across positive control outcomes, consistent with the less consistent evidence from the primary GCG-GLP1R analyses.” |
|  | c) | Report any assessment of direction of causal relationship (e.g., bidirectional MR) | / | / |
|  | d) | When relevant, report and compare with estimates from non-MR analyses | / | / |
|  | e) | Consider additional plots to visualize results (e.g., leave-one-out analyses) | Fig4 | Fig4 |
|  | **DISCUSSION** |  |  |  |
| 14 | **Key results** | Summarize key results with reference to study objectives | 17 | “In this human genetic study, we addressed a major safety question surrounding incretin-based therapies by evaluating whether perturbation of incretin signalling is associated with fracture risk. We observed an association between genetically proxied GIP-GIPR pathway perturbation and lower fracture risk, whereas evidence for GCG-GLP1R was less consistent. These findings were supported by several complementary analyses and differed from patterns observed for genome-wide liability to lower BMI or HbA1c.” |
| 15 | **Limitations** | Discuss limitations of the study, taking into account the validity of the IV assumptions, other sources of potential bias, and imprecision. Discuss both direction and magnitude of any potential bias and any efforts to address them | 18 | This study has several limitations. Fracture outcomes were defined using healthcare records and may include a mixture of low- and high-trauma events, although such definitions capture relevant genetic susceptibility.29 Multiple strategies were used to assess pleiotropic bias, but these cannot fully exclude horizontal pleiotropy or establish that the observed associations are mediated specifically through the GIP-GIPR pathway, consistent with the inherent limitations of Mendelian randomization. Genetic proxies represent lifelong pathway perturbation and may not reflect the magnitude, timing, or pharmacokinetics of therapeutic exposure, particularly given the higher potency and sustained duration of pharmacological compared with endogenous incretin signalling. For GIPR, human genetic studies have identified variants associated with lower BMI that alter or reduce receptor function, whereas pharmacological GIPR agonism produces weight loss through receptor activation. This difference in molecular mechanism emphasises that our findings cannot be directly extrapolated to fracture outcomes under GIPR agonist treatment, and should be interpreted as reflecting pathway perturbation rather than direct pharmacological effects. Finally, the main analyses were conducted in White British participants, which may limit generalisability to other ancestral groups. |
| 16 | **Interpretation** |  |  |  |
|  | a) | Meaning: Give a cautious overall interpretation of results in the context of their limitations and in comparison with other studies | 17-18 | These results provide evidence relevant to the skeletal safety of incretin-based therapies, a question that remains unresolved despite widespread clinical use.5, 6 Randomized trials have generally not been powered for fracture outcomes,7, 8 and observational studies have reported mixed findings but are susceptible to confounding and biases related to weight loss and healthcare utilization.9-11 Our findings provide complementary evidence from a human genetic framework that is less vulnerable to these biases.  The association pattern for GIP-GIPR perturbation differed from that observed for genome-wide genetic liability to lower BMI or HbA1c. Genetic liability to lower BMI was associated with higher fracture risk and lower bone density, consistent with established links between weight loss and skeletal fragility.38, 39 In contrast, GIP-GIPR perturbation was associated with lower fracture risk despite being scaled to BMI and HbA1c. This difference suggests that the observed GIP-GIPR association does not simply recapitulate the skeletal pattern of generalized lower-BMI liability, a finding consistent with a recent target-trial emulation in which GLP-1 receptor agonist initiation was associated with lower fragility-fracture risk not explained by changes in BMI or HbA1c.40 However, these comparisons do not establish independence from BMI or HbA1c, nor do they identify a specific biological mechanism underlying the association.  We did not observe an association between the primary GIP-GIPR pathway-level instruments and estimated bone mineral density captured by calcaneal ultrasound. Importantly, this finding does not allow us to determine whether GIP-GIPR perturbation influences other determinants of fracture risk, as this study could not directly assess bone microarchitecture, muscle mass, trauma mechanism, or other non-BMD determinants of fracture risk. Further studies incorporating imaging-based bone phenotypes and functional outcomes will be important to clarify these mechanisms.  Evidence for the GCG-GLP1R pathway was less consistent, with protective associations for spine fracture not uniformly supported across control outcomes and sensitivity analyses. These results suggest that inference for GLP1-related pathways is more sensitive to instrument definition. Accordingly, these analyses were treated as exploratory. |
|  | b) | Mechanism: Discuss underlying biological mechanisms that could drive a potential causal relationship between the investigated exposure and the outcome, and whether the gene-environment equivalence assumption is reasonable. Use causal language carefully, clarifying that IV estimates may provide causal effects only under certain assumptions | 18 | These results provide evidence relevant to the skeletal safety of incretin-based therapies, a question that remains unresolved despite widespread clinical use.5, 6 Randomized trials have generally not been powered for fracture outcomes,7, 8 and observational studies have reported mixed findings but are susceptible to confounding and biases related to weight loss and healthcare utilization.9-11 Our findings provide complementary evidence from a human genetic framework that is less vulnerable to these biases.  The association pattern for GIP-GIPR perturbation differed from that observed for genome-wide genetic liability to lower BMI or HbA1c. Genetic liability to lower BMI was associated with higher fracture risk and lower bone density, consistent with established links between weight loss and skeletal fragility.38, 39 In contrast, GIP-GIPR perturbation was associated with lower fracture risk despite being scaled to BMI and HbA1c. This difference suggests that the observed GIP-GIPR association does not simply recapitulate the skeletal pattern of generalized lower-BMI liability, a finding consistent with a recent target-trial emulation in which GLP-1 receptor agonist initiation was associated with lower fragility-fracture risk not explained by changes in BMI or HbA1c.40 However, these comparisons do not establish independence from BMI or HbA1c, nor do they identify a specific biological mechanism underlying the association.  We did not observe an association between the primary GIP-GIPR pathway-level instruments and estimated bone mineral density captured by calcaneal ultrasound. Importantly, this finding does not allow us to determine whether GIP-GIPR perturbation influences other determinants of fracture risk, as this study could not directly assess bone microarchitecture, muscle mass, trauma mechanism, or other non-BMD determinants of fracture risk. Further studies incorporating imaging-based bone phenotypes and functional outcomes will be important to clarify these mechanisms.  Evidence for the GCG-GLP1R pathway was less consistent, with protective associations for spine fracture not uniformly supported across control outcomes and sensitivity analyses. These results suggest that inference for GLP1-related pathways is more sensitive to instrument definition. Accordingly, these analyses were treated as exploratory.  Sex-stratified estimates were more precise in females than males, potentially reflecting differences in fracture event counts and statistical power, and should not be interpreted as evidence of sex-specific effect modification. |
|  | c) | Clinical relevance: Discuss whether the results have clinical or public policy relevance, and to what extent they inform effect sizes of possible interventions | 19 | “In summary, genetic evidence suggests an association between genetically proxied GIP-GIPR pathway perturbation and lower fracture risk. These findings should not be interpreted as evidence that pharmacological GIPR agonism reduces fracture risk, but support fracture-focused evaluation of GIPR-targeting therapies in clinical and post-marketing studies.” |
| 17 | **Generalizability** | Discuss the generalizability of the study results (a) to other populations, (b) across other exposure periods/timings, and (c) across other levels of exposure | 18 | “Finally, the main analyses were conducted in White British participants, which may limit generalisability to other ancestral groups.”  “Genetic proxies represent lifelong pathway perturbation and may not reflect the magnitude, timing, or pharmacokinetics of therapeutic exposure, particularly given the higher potency and sustained duration of pharmacological compared with endogenous incretin signalling. For GIPR, human genetic studies have identified variants associated with lower BMI that alter or reduce receptor function, whereas pharmacological GIPR agonism produces weight loss through receptor activation. This difference in molecular mechanism emphasises that our findings cannot be directly extrapolated to fracture outcomes under GIPR agonist treatment, and should be interpreted as reflecting pathway perturbation rather than direct pharmacological effects.” |
|  | **OTHER INFORMATION** |  |  |  |
| 18 | **Funding** | Describe sources of funding and the role of funders in the present study and, if applicable, sources of funding for the databases and original study or studies on which the present study is based | 21 | SSZ is supported by an Arthritis UK Career Development Fellowship (grant no. 23258), and works in centres supported by Arthritis UK (grant no. 21173, 21754 and 21755) and the NIHR Manchester Biomedical Research Centre (NIHR203308). |
| 19 | **Data and data sharing** | Provide the data used to perform all analyses or report where and how the data can be accessed, and reference these sources in the article. Provide the statistical code needed to reproduce the results in the article, or report whether the code is publicly accessible and if so, where | sup | supplementary tableS1-23 |
| 20 | **Conflicts of Interest** | All authors should declare all potential conflicts of interest | 20 | SSZ has received support for conference attendance from Novartis, UCB, AlfaSigma, Eli Lilly; and consultancy/speaker fees from Novartis, UCB, AlfaSigma, AbbVie, Jassen, Khanda Therapeutics |

This checklist is copyrighted by the Equator Network under the Creative Commons Attribution 3.0 Unported (CC BY 3.0) license.

1.	Skrivankova VW, Richmond RC, Woolf BAR, Yarmolinsky J, Davies NM, Swanson SA, et al. Strengthening the Reporting of Observational Studies in Epidemiology using Mendelian Randomization (STROBE-MR) Statement. JAMA. 2021;under review.

2.	Skrivankova VW, Richmond RC, Woolf BAR, Davies NM, Swanson SA, VanderWeele TJ, et al. Strengthening the Reporting of Observational Studies in Epidemiology using Mendelian Randomisation (STROBE-MR): Explanation and Elaboration. BMJ. 2021;375:n2233.
